# Ketogenesis as a Metabolic Checkpoint in MASLD: Implications for Disease Progression and Therapy

**DOI:** 10.3390/metabo16090659

**Published:** 2026-09-08

**Authors:** Ambrin Farizah Babu

**Affiliations:** 1 Food Sciences Unit, Department of Life Technologies, University of Turku, 20014 Turku, Finland; ambrin.babu@utu.fi; 2 School of Medicine, Institute of Public Health and Clinical Nutrition, University of Eastern Finland, 70211 Kuopio, Finland

**Keywords:** metabolic dysfunction–associated steatotic liver disease (MASLD), ketogenesis, β-hydroxybutyrate, mitochondrial dysfunction, fatty acid oxidation, metabolic flexibility, lipotoxicity

## Abstract

Metabolic dysfunction-associated steatotic liver disease (MASLD) is the most common chronic liver disease worldwide, encompassing a spectrum from simple steatosis to metabolic dysfunction-associated steatohepatitis (MASH), fibrosis, cirrhosis and hepatocellular carcinoma. Increasing evidence indicates that disease progression is driven not by hepatic triglyceride accumulation alone but by the metabolic partitioning of excess fatty acids between adaptive and maladaptive pathways. Ketogenesis, traditionally viewed as a fasting-induced mechanism for disposing of excess acetyl-CoA, is now recognized as a key regulator of hepatic metabolic homeostasis, coordinating mitochondrial substrate utilization, carbon flux and systemic metabolic adaptation. In addition to serving as oxidative fuels, ketone bodies, particularly β-hydroxybutyrate, function as signalling metabolites that modulate inflammation, oxidative stress, mitochondrial function and epigenetic regulation. Despite increased fatty acid delivery in obesity and insulin resistance, ketogenic capacity becomes progressively impaired during MASLD, promoting mitochondrial acetyl-CoA accumulation, oxidative stress and diversion of carbon toward lipotoxic lipid synthesis while reducing protective β-hydroxybutyrate signalling. This review examines ketogenesis as an integrative metabolic checkpoint linking fatty acid oxidation, lipid metabolism, mitochondrial function and immune signalling in MASLD. We discuss how impaired ketogenic flux contributes to hepatocellular injury, fibrosis and metabolic inflexibility, and evaluate the therapeutic potential of restoring ketogenesis to prevent disease progression.

## 1. Introduction

Metabolic dysfunction-associated steatotic liver disease (MASLD), previously known as non-alcoholic fatty liver disease (NAFLD), has become the leading cause of chronic liver disease worldwide, mirroring the global rise in obesity, insulin resistance, type 2 diabetes mellitus, and metabolic syndrome [1]. The disease encompasses a broad pathological spectrum ranging from isolated steatosis to metabolic dysfunction-associated steatohepatitis (MASH), progressive fibrosis, cirrhosis, and hepatocellular carcinoma [2]. Although hepatic steatosis is the defining feature of MASLD, only a minority of affected individuals progress to advanced liver disease, indicating that lipid accumulation alone is insufficient to explain hepatocellular injury [3].

MASLD is increasingly recognized as a disease of disrupted metabolic adaptation. Under physiological conditions, hepatocytes continuously coordinate fatty acid uptake, oxidation, storage and export to maintain metabolic homeostasis despite substantial fluctuations in nutrient availability [4]. During chronic nutrient excess, however, this adaptive capacity becomes progressively impaired. The resulting loss of metabolic flexibility alters intracellular carbon partitioning, promotes mitochondrial stress, enhances lipotoxicity and ultimately initiates inflammatory and fibrogenic responses that drive disease progression [5,6].

Historically, research has focused predominantly on mitochondrial β-oxidation as the major determinant of hepatic lipid disposal. Impaired β-oxidation has long been proposed to promote triglyceride accumulation, oxidative stress, and mitochondrial dysfunction in MASLD [3,7]. However, this paradigm only partially explains disease pathogenesis. Hepatic triglyceride accumulation is necessary for the diagnosis of MASLD but is insufficient to explain disease progression. In well-characterized clinical cohorts, histological activity plateaus despite further increases in intrahepatic triglyceride content, indicating that the transition from simple steatosis to steatohepatitis and fibrosis is not driven by the quantity of stored triglyceride per se [8]. Studies suggest that triglyceride synthesis is not intrinsically pathogenic but instead constitutes an adaptive mechanism that protects hepatocytes by esterifying and sequestering excess fatty acids within lipid droplets, thereby limiting the accumulation of lipotoxic lipid intermediates [9]. These observations suggest that the critical determinant of disease progression is not the amount of lipid stored within the liver, but the metabolic fate of excess fatty acids.

Among the metabolic pathways governing fatty acid disposal, hepatic fatty acids can undergo several interconnected fates, including mitochondrial β-oxidation, triglyceride synthesis and storage in lipid droplets, very low-density lipoprotein (VLDL) secretion, and the generation of bioactive lipid species [10]. Within this network, ketogenesis provides a mitochondrial route for diverting fatty acid-derived acetyl-CoA toward the production of ketone bodies acetoacetate, β-hydroxybutyrate (βHB), and acetone [11]. Traditionally regarded as a metabolic adaptation to fasting or starvation, ketogenesis has largely been considered irrelevant to diseases of nutrient excess. However, accumulating evidence challenges this view. Ketogenesis prevents mitochondrial acetyl-CoA accumulation and supports continued fatty acid oxidation. Further, it also produces βHB, a signalling metabolite capable of regulating inflammation, oxidative stress, mitochondrial function and epigenetic programmes [12,13]. These discoveries have expanded the role of ketogenesis from a simple energy-producing pathway to a central regulator of hepatic metabolic homeostasis.

Ketogenesis is closely connected with other pathways involved in fatty acid metabolism. Hepatic fatty acids are continuously partitioned between oxidative pathways, triglyceride synthesis, lipid droplet turnover, VLDL secretion and the synthesis of bioactive lipids such as ceramides and diacylglycerols [14,15]. These pathways compete for common metabolic substrates while reciprocally regulating one another. Perturbations in ketogenic flux therefore have consequences that extend throughout the hepatic lipid network, influencing mitochondrial function, cellular signalling and inflammatory responses. Conversely, alterations in non-oxidative lipid metabolism can impair mitochondrial function and further suppress ketogenesis [15], establishing a self-amplifying cycle of metabolic dysfunction.

This study reviews how ketogenesis influences hepatic adaptation to lipid excess in MASLD. We discuss how ketogenic insufficiency contributes to metabolic inflexibility, lipotoxicity, and mitochondrial dysfunction in MASLD, examine the signalling functions of βHB, and explore how interactions between ketogenesis and non-oxidative lipid pathways shape inflammation, fibrosis, and disease progression. Understanding these interconnected metabolic networks may provide a more comprehensive framework for MASLD pathogenesis and identify new opportunities for therapeutic intervention.

Relevant literature was identified through searches of PubMed/MEDLINE, Web of Science and Scopus during preparation of this review. Search terms included combinations of “ketogenesis”, “ketone bodies”, “β-hydroxybutyrate”, “HMGCS2”, “fatty acid oxidation”, “autophagy”, “lipophagy”, “insulin resistance”, “hepatic macrophages”, “inflammation”, “fatty liver”, “fibrosis”, “MASLD”, “NAFLD”, “NASH”, and “MASH”. We prioritized original human and experimental studies, supported by relevant mechanistic and review articles, that addressed hepatic ketogenesis, metabolic adaptation, or their relationship to MASLD pathogenesis and therapeutic strategies. Reference lists of key publications were also screened to identify additional relevant studies.

## 2. Ketogenesis as a Central Regulator of Hepatic Metabolic Homeostasis

Ketogenesis has traditionally been viewed as a hepatic adaptation to prolonged fasting, functioning primarily to provide alternative oxidative substrates for peripheral tissues when glucose availability is limited. Recent advances in metabolism have substantially expanded this perspective. Ketogenesis is increasingly recognized as a fundamental component of hepatic metabolic homeostasis, integrating fatty acid oxidation, mitochondrial function, redox regulation, and inter-organ communication [16,17,18,19,20,21,22,23]. By converting excess acetyl-CoA into ketone bodies, ketogenesis enables continued mitochondrial β-oxidation when fatty acid supply exceeds tricarboxylic acid (TCA) cycle capacity [16]. The liver occupies a unique position in systemic ketone metabolism. Hepatocyte mitochondria are the primary site of ketone body synthesis, whereas extrahepatic tissues including skeletal muscle, heart, kidney and brain oxidize ketone bodies to generate adenosine triphosphate (ATP) [11]. Consequently, ketogenesis enables the export of excess carbon derived from hepatic fatty acid oxidation, thereby coordinating energy metabolism across multiple organs.

Ketogenesis occurs when mitochondrial β-oxidation produces excess acetyl-CoA beyond TCA cycle capacity, driving its conversion into acetoacetate, which is then reduced to βHB or decarboxylated to acetone, with the βHB/acetoacetate ratio determined by the mitochondrial NADH/NAD^+^ redox state [11]. Ketogenic flux is tightly controlled by hormonal, nutritional and transcriptional mechanisms [24]. During fasting, declining insulin concentrations and increased glucagon stimulate adipose tissue lipolysis, increasing hepatic fatty acid delivery and activating ketogenesis. At the transcriptional level, peroxisome proliferator-activated receptor-α (PPARα) serves as the master regulator of hepatic ketogenesis by inducing 3-hydroxy-3-methylglutaryl-CoA synthase 2 (HMGCS2) and numerous genes involved in mitochondrial fatty acid oxidation. Additional transcription factors, including forkhead box protein A2 (FOXA2), hepatocyte nuclear factor 4 alpha (HNF4α) and Peroxisome proliferator-activated receptor gamma coactivator 1-alpha (PGC-1α), integrate nutritional status with mitochondrial metabolic capacity, ensuring that ketogenesis responds appropriately to changes in substrate availability. Mitochondrial acetyl-CoA concentrations, free coenzyme A availability and cellular redox state further modulate ketogenic activity, enabling rapid metabolic adaptation [11].

Ketone bodies, particularly βHB, are now recognized to function as signalling metabolites in addition to serving as energy substrates. βHB influences cellular physiology through diverse mechanisms, including inhibition of class I histone deacetylases, regulation of lysine β-hydroxybutyrylation, activation of G-protein-coupled receptors and modulation of intracellular signalling pathways [25]. Through these mechanisms, βHB regulates transcriptional programmes governing oxidative stress resistance, mitochondrial biogenesis, autophagy and cellular repair. In parallel, βHB suppresses activation of the NLRP3 inflammasome and influences macrophage inflammatory responses, establishing ketogenesis as an important component of immunometabolic regulation [26]. These signalling functions position ketone bodies as endocrine mediators that communicate hepatic metabolic status to both intrahepatic and extrahepatic tissues.

These findings challenge the long-held view that ketogenesis is restricted to the physiological response to fasting. Ketogenesis appears to be a central metabolic process that coordinates mitochondrial substrate utilisation with cellular signalling, redox homeostasis, and systemic metabolic adaptation. This broader perspective provides a conceptual foundation for understanding why disruption of ketogenic flux may have profound consequences during chronic nutrient excess. In MASLD, where mitochondrial fatty acid oxidation, lipid partitioning and inflammatory signalling become progressively dysregulated [3], impairment of this adaptive pathway may contribute not only to defective energy metabolism but also to lipotoxicity, mitochondrial dysfunction and chronic liver injury. The following sections examine how this ketogenic insufficiency develops in MASLD and how it reshapes hepatic lipid metabolism to drive disease progression.

## 3. Ketogenic Insufficiency in Metabolic Dysfunction—Associated Steatotic Liver Disease

**Figure 1:**
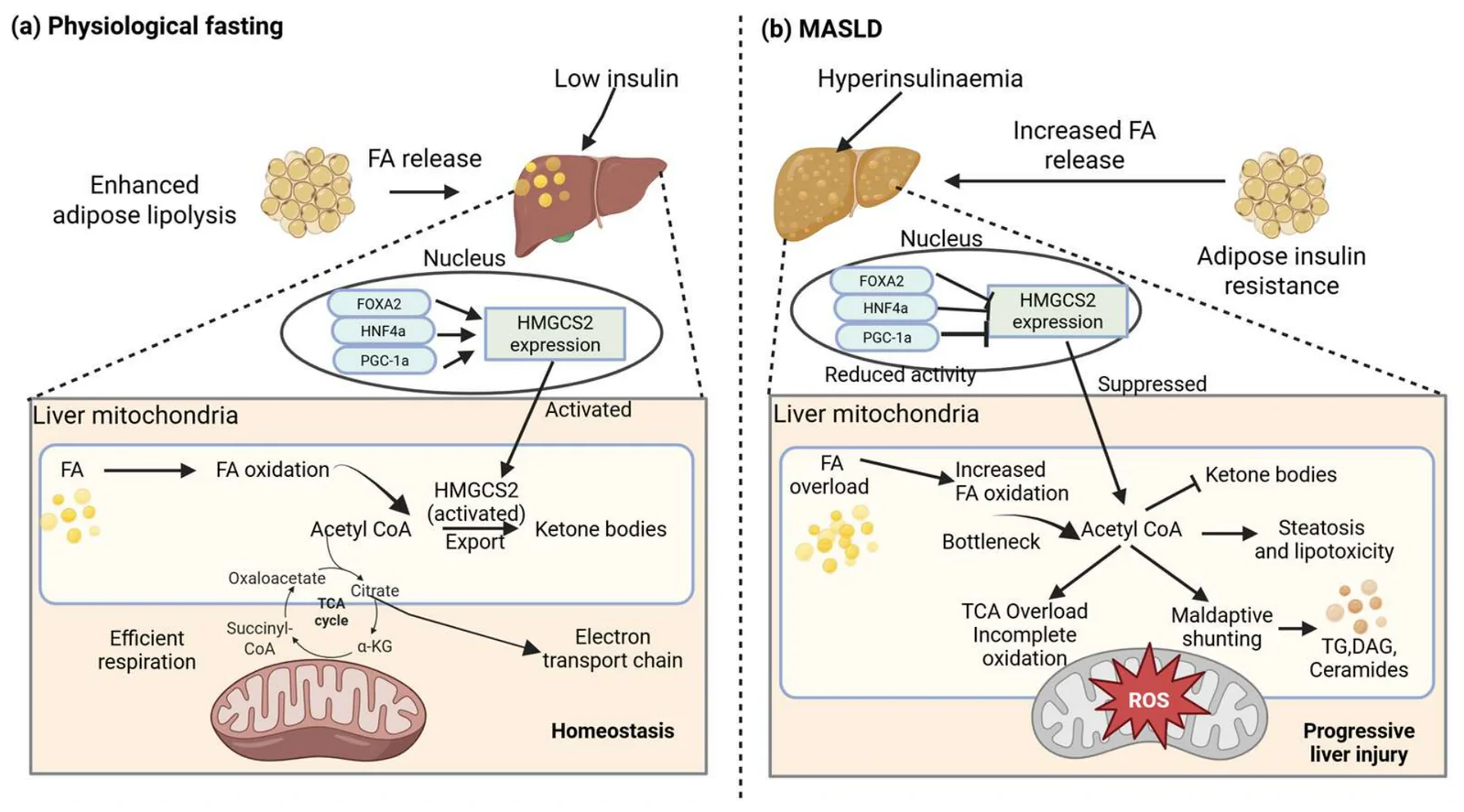
Comparative metabolic pathways of hepatic fatty acid flux in (**a**) physiological states vs. (**b**) metabolic dysfunction-associated steatotic liver disease (MASLD). FA—fatty acid, ROS—Reactive oxygen species, FOXA2—Forkhead Box A2, PGC1a—Peroxisome proliferator-activated receptor gamma coactivator 1-alpha, HNF4a—Hepatocyte Nuclear Factor 4 alpha, HMGCS2—Hydroxymethylglutaryl-CoA synthase 2. Created in BioRender. Babu, A. (2026) https://BioRender.com/qerzipo.

In MASLD, hepatic fatty acid delivery is increased, but ketone body production does not increase proportionally, suggesting impaired adaptation to lipid oversupply. Under physiological fasting conditions, enhanced fatty acid oxidation is accompanied by a proportional increase in ketogenesis, allowing excess acetyl-CoA to be exported from the liver (Figure 1a). In obesity and insulin resistance, however, fatty acid oversupply is accompanied by a comparatively blunted ketogenic response [27], suggesting altered partitioning of fatty acid-derived acetyl-CoA rather than limited substrate availability (Figure 1b). Importantly, evidence from human studies supports an association between impaired ketogenic flexibility and metabolic dysfunction. Individuals with obesity and insulin resistance exhibit altered ketone-body responses to fasting or lipid provision, consistent with impaired adaptation of hepatic ketogenesis to increased fatty acid availability [28,29,30]. Moreover, metabolic interventions that improve insulin sensitivity and hepatic metabolic flexibility can modify ketone-body production (reviewed in [31], supporting a functional relationship between systemic metabolic status and ketogenic capacity.

Although β-oxidation has traditionally been considered impaired in MASLD, more recent evidence indicates a more nuanced metabolic adaptation. Recent studies suggest that mitochondrial fatty acid oxidation is initially increased in response to lipid overload, reflecting an attempt to compensate for excessive fatty acid influx [32]. However, this adaptive response is ultimately unable to match the magnitude of lipid delivery. Moreover, increased β-oxidation does not necessarily translate into efficient downstream metabolism. Instead, mitochondrial substrate overload, impaired respiratory efficiency and reduced metabolic flexibility may limit the capacity to channel acetyl-CoA into ketogenesis, possibly resulting in incomplete fatty acid oxidation and progressive mitochondrial stress.

Clinical studies further suggest that the relationship between ketogenesis and MASLD is dependent on metabolic context and disease stage. Circulating βHB concentrations are not consistently reduced in individuals with obesity or MASLD; in some cohorts they are unchanged or even increased [33,34]. This apparent discrepancy does not necessarily contradict the concept of ketogenic insufficiency, because circulating ketone concentrations reflect the dynamic balance between hepatic production and peripheral utilization rather than hepatic ketogenic flux alone [12]. In insulin resistance, increased fatty acid delivery and changes in peripheral ketone utilization may mask a reduced ability of the liver to increase ketogenesis when metabolic demand rises [33]. Therefore, the relevant phenotype may not be simply low circulating ketone concentrations, but impaired ketogenic flexibility (i.e., the capacity to adjust hepatic ketone production to changes in fatty acid availability).

Overall, the available evidence links impaired ketogenic adaptation with the metabolic abnormalities commonly seen in MASLD, including obesity, insulin resistance, and altered hepatic lipid metabolism. Whether this impairment is associated with MASLD severity or fibrosis is still unclear. Circulating ketone concentrations alone may not be sufficient to answer this question, since they are also influenced by peripheral ketone utilization. Assessing hepatic ketone production directly may therefore provide a better indication of whether defects in ketogenesis contribute to disease progression.

### 3.1. Mechanisms Underlying Impaired Ketogenesis

Multiple mechanisms likely contribute to the suppression of hepatic ketogenesis during metabolic dysfunction. Insulin resistance may play an important role in impaired ketogenic adaptation in MASLD, as hepatic fatty acid availability can remain elevated despite persistent hyperinsulinaemia [35]. Hyperinsulinaemia, a defining feature of insulin-resistant states, directly inhibits the ketogenic programme by suppressing the expression and activity of mitochondrial 3-hydroxy-3-methylglutaryl-CoA synthase (HMGCS2), the rate-limiting enzyme of ketone body synthesis [19,36]. At the same time, insulin suppresses adipose tissue lipolysis under physiological conditions; however, adipose insulin resistance results in continued fatty acid release despite persistent hyperinsulinaemia [37]. Consequently, insulin resistance generates a mismatch between increased fatty acid delivery to the liver and impaired activation of hepatic ketogenesis. This mismatch may promote diversion of fatty acid-derived carbon towards triglyceride synthesis and potentially lipotoxic lipid species.

Transcriptional dysregulation further contributes to reduced ketogenic capacity. Peroxisome proliferator-activated receptor-α (PPARα), a key regulator of hepatic fatty acid oxidation and ketogenesis, exhibits altered activity during MASLD progression [38]. Reduced expression of PPARα target genes, including *HMGCS2*, limits the coordinated induction of the ketogenic pathway despite increased lipid availability [39]. Additional alterations in FOXA2, PGC-1α and fibroblast growth factor 21 (FGF21) signalling further impair the transcriptional network required to maintain mitochondrial metabolic flexibility [40,41,42].

Mitochondrial dysfunction provides another important constraint on ketogenesis. Progressive impairment of mitochondrial respiration, alterations in the NADH/NAD^+^ ratio, decreased coenzyme A availability and structural mitochondrial abnormalities all reduce the efficiency of acetyl-CoA utilization [43,44]. Because ketogenesis is highly dependent on mitochondrial redox status, disturbances in oxidative phosphorylation may limit ketone body production even when acetyl-CoA concentrations are elevated. Insulin resistance may therefore act upstream of multiple defects in the ketogenic pathway, increasing fatty acid delivery while simultaneously limiting the capacity of hepatocytes to dispose of fatty acid-derived carbon through ketogenesis. This metabolic uncoupling may contribute to mitochondrial stress, altered lipid partitioning and progressive hepatic injury.

### 3.2. Ketogenic Insufficiency as a Driver of Lipotoxicity

The consequences of reduced ketogenic flux extend beyond diminished ketone body production. Ketogenesis normally functions as a mitochondrial overflow pathway, exporting excess acetyl-CoA as ketone bodies when fatty acid oxidation exceeds the oxidative capacity of the TCA cycle [11]. When this adaptive mechanism becomes constrained, acetyl-CoA accumulates within mitochondria, increasing flux through the TCA cycle and driving excessive electron donation to the electron transport chain. The resulting increase in reactive oxygen species (ROS) production promotes oxidative damage to mitochondrial proteins, lipids and deoxyribonucleic acid (DNA), contributing to the progressive decline in mitochondrial function characteristic of MASLD [45].

Accumulation of mitochondrial acetyl-CoA may influence intracellular carbon partitioning by altering the balance between oxidative disposal, ketogenesis, and lipid synthesis pathways. When ketogenic capacity is impaired, excess fatty acid-derived carbon may contribute to increased synthesis of non-oxidative lipid species, including triglycerides [33]. This shift may promote lipotoxic stress and help explain why hepatocellular injury correlates poorly with total hepatic triglyceride accumulation. Rather than the absolute amount of stored lipid, the metabolic fate of fatty acids and the balance between adaptive lipid storage and harmful lipid signaling pathways appear to be key determinants of hepatic injury [46].

Importantly, the consequences of impaired ketogenesis may extend beyond hepatocellular lipotoxicity to influence the development of hepatic fibrosis. Reduced ketogenic flux may increase mitochondrial ROS and the accumulation of lipotoxic lipid species, thereby promoting hepatocyte stress, apoptosis and the release of damage-associated molecular patterns and inflammatory mediators (Reviewed in [47]). These signals can activate hepatic macrophages and amplify inflammatory pathways, including transforming growth factor-β (TGF-β) signalling, which promotes activation of hepatic stellate cells and their transition towards an extracellular-matrix-producing phenotype. Persistent stellate-cell activation and excessive extracellular matrix deposition provide a plausible mechanistic link between impaired ketogenic adaptation and progressive hepatic fibrosis [48].

Evidence from experimental models supports this concept. Hepatic deletion of *Hmgcs2* exacerbates steatosis, oxidative stress, inflammation and fibrosis despite ongoing fatty acid oxidation, whereas restoration of ketogenic capacity improves mitochondrial function and hepatic metabolic flexibility [19]. These findings suggest that preservation of ketogenic flux may limit fibrogenesis by reducing mitochondrial stress and downstream inflammatory signalling, although the direct contribution of impaired ketogenesis to fibrosis in human MASLD remains incompletely established. Human studies are less consistent, with reports of both increased and reduced circulating ketone body concentrations in obesity and MASLD [49,50]. However, circulating ketone levels represent the balance between hepatic production and peripheral utilization rather than direct measurements of hepatic ketogenic flux. Differences in disease stage, fasting duration, insulin sensitivity and substrate availability likely explain much of the apparent discrepancy across studies, suggesting that the capacity to appropriately regulate ketogenesis, rather than absolute ketone body concentrations, may be the critical determinant of hepatic metabolic health.

## 4. Ketogenesis and Non-Oxidative Lipid Metabolism

The metabolic fate of hepatic fatty acids is determined by a complex network of interconnected pathways rather than by isolated enzymatic reactions. Fatty acids entering hepatocytes may undergo mitochondrial β-oxidation, be incorporated into triglycerides for storage within lipid droplets, be packaged into very-low-density lipoproteins for secretion, or serve as substrates for the synthesis of bioactive lipid species [51]. These pathways compete for common substrates while simultaneously regulating one another through reciprocal metabolic and signalling interactions. Consequently, alterations in ketogenic flux inevitably reshape the distribution of intracellular lipid metabolism, influencing both adaptive and pathogenic responses to nutrient excess.

### 4.1. Triglyceride Synthesis: A Protective Adaptation with Finite Capacity

Previously, triglyceride accumulation was considered the principal pathogenic event in MASLD. This view has been challenged by growing evidence that triglyceride synthesis represents, at least initially, a protective adaptation. Esterification of free fatty acids into triglycerides reduces the intracellular concentration of lipotoxic lipid species and enables their sequestration within lipid droplets, thereby limiting membrane disruption, endoplasmic reticulum stress and mitochondrial injury [52]. Consistent with this concept, experimental inhibition of triglyceride synthesis often worsens hepatocellular injury despite reducing hepatic fat accumulation [53], whereas enhanced triglyceride storage may attenuate lipotoxicity under certain conditions.

This protective mechanism, however, has finite capacity. Persistent lipid oversupply eventually overwhelms triglyceride buffering, leading to excessive lipid droplet expansion, impaired turnover and altered intracellular lipid trafficking [51]. At this stage, triglyceride accumulation no longer reflects effective metabolic adaptation but rather an inability of hepatocytes to appropriately process excess lipid. Thus, hepatic steatosis should be viewed as a dynamic marker of altered lipid partitioning rather than the primary cause of cellular injury.

### 4.2. Lipid Droplet Dynamics and Organelle Crosstalk

Lipid droplets are increasingly recognized as dynamic metabolic organelles that actively regulate intracellular lipid homeostasis. In addition to functioning as inert depots, they continuously exchange lipids with mitochondria, the endoplasmic reticulum, lysosomes and peroxisomes through coordinated processes of triglyceride synthesis, lipolysis and lipophagy [54]. These interactions determine the availability of fatty acids for mitochondrial β-oxidation and ketogenesis while simultaneously preventing excessive accumulation of free fatty acids within the cytoplasm.

Disruption of lipid droplet dynamics profoundly alters mitochondrial metabolism. Impaired lipophagy limits the controlled release of fatty acids required for efficient mitochondrial oxidation, whereas excessive or dysregulated lipolysis may expose mitochondria to sudden surges of fatty acid flux that exceed their oxidative capacity [55]. In both situations, mitochondrial metabolic flexibility is compromised. Because ketogenesis depends on a tightly regulated supply of acetyl-CoA and intact mitochondrial function, disturbances in lipid droplet turnover may indirectly suppress ketogenic flux, further exacerbating metabolic dysfunction.

### 4.3. Autophagy Links Lipid Mobilization to Ketogenesis

Autophagy links hepatic lipid droplet turnover with ketone production. Through lipophagy, triglycerides stored in lipid droplets are broken down and the released fatty acids can be delivered to mitochondria for β-oxidation and ketogenesis [56]. Experimental studies in mice have shown that loss of hepatic autophagy attenuates the increase in ketone production during fasting, with lower β-hydroxybutyrate levels and reduced hepatic ketone content despite preserved induction of the ketogenic enzyme HMG-CoA synthase 2 [57]. Autophagy therefore appears to support ketogenesis during fasting through effects on hepatic lipid metabolism and fatty acid oxidation.

This process may be altered in MASLD, where impaired autophagic flux and changes in lipid droplet turnover could affect fatty acid delivery to mitochondria [58]. Reduced lipophagy may limit the supply of fatty acids for ketogenesis. Conversely, when fatty acid mobilization exceeds mitochondrial oxidative capacity, it may contribute to lipid accumulation and cellular stress. β-HB has also been implicated in the regulation of autophagy [25], suggesting that changes in ketone production may influence these processes as well.

### 4.4. Bioactive Lipid Signalling and Metabolic Reprogramming

When oxidative pathways become saturated or dysfunctional, excess fatty acids are increasingly diverted into the synthesis of bioactive lipid intermediates, including ceramides, diacylglycerols, lysophospholipids and oxidized lipid species [15]. Unlike triglycerides, these lipids are potent signalling molecules capable of reprogramming cellular metabolism. Ceramides inhibit insulin signalling, impair mitochondrial respiration and promote apoptosis [59], whereas diacylglycerols activate novel protein kinase C isoforms that exacerbate hepatic insulin resistance [60]. Oxidized phospholipids and lysophospholipids further amplify inflammatory signalling, endoplasmic reticulum stress and hepatocyte death [61].

The production of these lipotoxic species is closely linked to ketogenic capacity. Efficient ketogenesis diverts acetyl-CoA away from anabolic pathways, facilitating continued fatty acid oxidation while reducing substrate availability for bioactive lipid synthesis [11]. Conversely, impaired ketogenesis increases intracellular carbon availability for non-oxidative lipid metabolism, favouring the accumulation of signalling lipids that promote inflammation and fibrosis [33]. This reciprocal relationship suggests that ketogenesis functions not merely as an endpoint of β-oxidation but as a critical determinant of intracellular lipid partitioning.

## 5. β-Hydroxybutyrate Signalling in MASLD: Linking Metabolism to Cellular Adaptation

The recognition that ketone bodies function as signalling molecules has fundamentally altered our understanding of ketogenesis. Traditionally viewed as alternative oxidative substrates that sustain extrahepatic energy metabolism during fasting, ketone bodies are now appreciated as pleiotropic regulators of gene expression, inflammation, redox homeostasis and cellular stress responses. Among the three ketone bodies, βHB has emerged as the principal mediator of these non-canonical functions. Consequently, the biological consequences of impaired ketogenesis extend beyond defective acetyl-CoA disposal and include the loss of endogenous signalling pathways that may protect the liver against metabolic injury.

βHB therefore has two distinct but interconnected biological roles. First, it serves as an alternative oxidative fuel during periods of limited glucose availability or prolonged fasting. Produced in the liver from fatty acids, βHB is transported to extrahepatic tissues, including the brain, heart, kidney, and skeletal muscle, where it is converted to acetyl-CoA and oxidized for ATP production [62,63,64,65]. Second, βHB acts as a signalling metabolite and can influence cellular processes through G protein-coupled receptors, ion channels, and epigenetic mechanisms, including inhibition of histone deacetylases and lysine β-hydroxybutyrylation [66]. These two functions may be relevant to MASLD, as impaired ketogenesis could reduce both the availability of βHB as an oxidative substrate and its signalling effects. At the same time, circulating βHB does not necessarily reflect hepatic ketogenesis alone, since its concentration is determined by the balance between hepatic production and peripheral utilization.

### 5.1. Regulation of Inflammation and Innate Immunity

Chronic low-grade inflammation is a defining feature of progressive MASLD and represents a major determinant of the transition from simple steatosis to MASH [7]. Hepatocyte injury induced by lipotoxicity promotes the release of damage-associated molecular patterns, which activate resident macrophages, recruit circulating immune cells and initiate inflammatory cascades that ultimately drive fibrogenesis [67]. Within this context, βHB has emerged as an endogenous regulator of innate immune responses [68], representing its signalling role.

**Figure 2:**
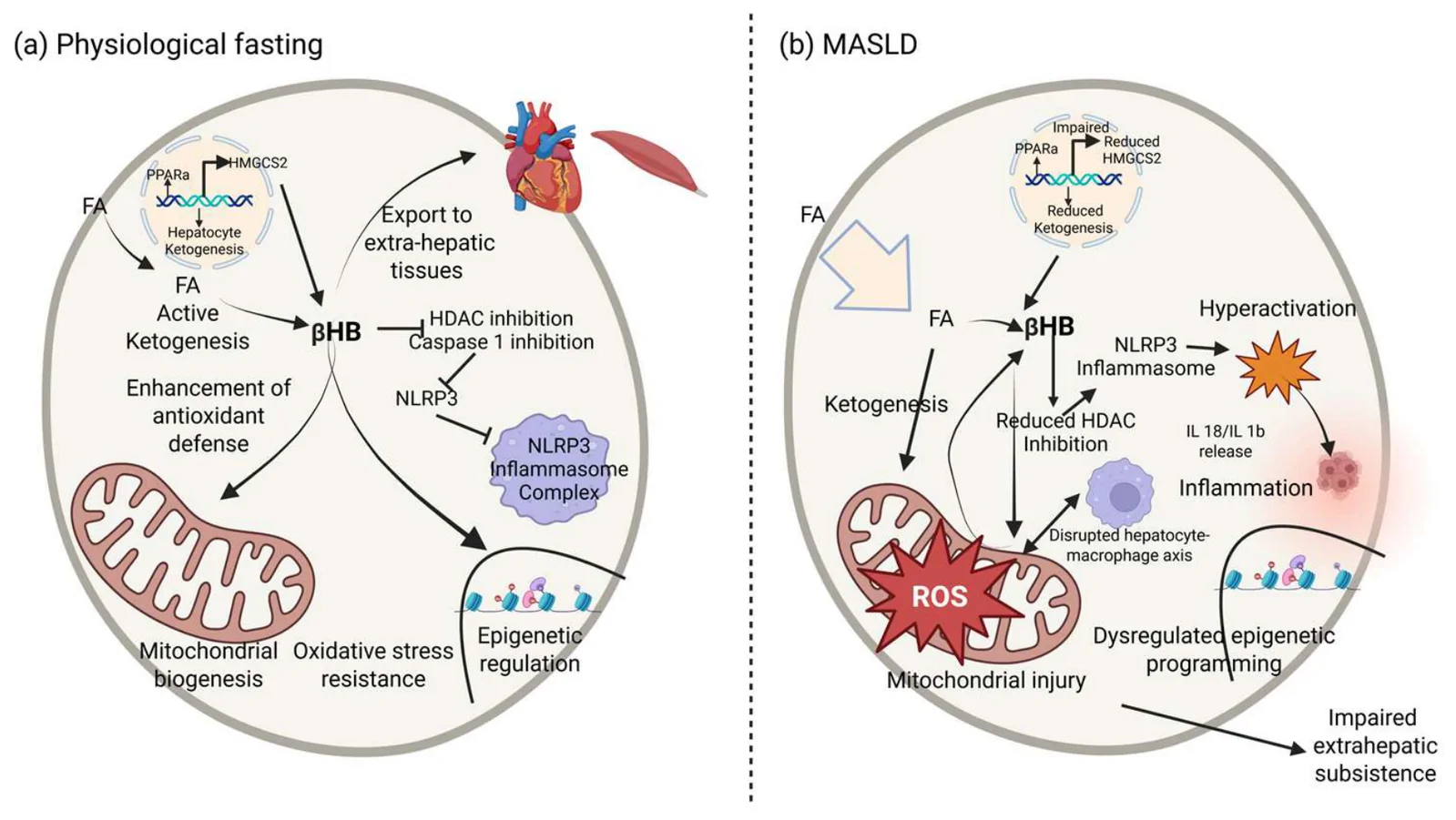
Dual Signaling Roles of βHB in Hepatic Homeostasis in (**a**) physiological states vs. (**b**) metabolic dysfunction-associated steatotic liver disease (MASLD). FA—fatty acid, ROS—Reactive oxygen species, HDAC—Histone deacetylase, NLRP3—NLR family pyrin domain containing 3, IL—Interleukin. Created in BioRender. Babu, A. (2026) https://BioRender.com/ti8oq4d.

The most extensively characterized anti-inflammatory action of βHB is the inhibition of the NLR family pyrin domain containing 3 (NLRP3) inflammasome, a multiprotein complex that integrates metabolic stress with inflammatory signalling [69]. As shown in Figure 2, activation of the NLRP3 inflammasome promotes caspase-1-dependent maturation of interleukin-1β (IL-1β) and interleukin-18 (IL-18), cytokines that contribute to hepatocellular injury, immune cell recruitment and hepatic fibrosis [70]. Previous studies have demonstrated that βHB suppresses NLRP3 activation independently of glucose availability or insulin signalling [26,69], suggesting that ketone bodies directly regulate inflammatory responses.

In addition to inflammasome inhibition, βHB influences additional inflammatory pathways. Evidence from experimental models indicates that βHB alters NF-κB signalling, cytokine production and macrophage polarization towards anti-inflammatory phenotypes [71,72]. These effects may be particularly relevant in MASLD, where hepatic macrophages undergo dynamic phenotypic changes during disease progression. However, much of the mechanistic evidence has been generated in immune cells or extrahepatic tissues, and whether physiological concentrations of βHB exert comparable immunomodulatory effects within the human liver remains incompletely understood. Defining the cell-specific actions of βHB within the hepatic immune microenvironment therefore represents an important priority for future investigation.

### 5.2. Hepatic Macrophages and Ketogenesis

Although hepatocytes are the principal site of ketogenesis, hepatic macrophages are increasingly recognized as regulators of the ketogenic response [47]. During fasting, glucocorticoid receptor signalling in hepatic macrophages restrains tumour necrosis factor-alpha (TNF-α) production and promotes glucocorticoid receptor activation in neighbouring hepatocytes, where it cooperates with PPARα to induce fatty acid oxidation and ketogenesis [73]. Therefore, macrophages can shape hepatic ketogenic flux through paracrine control of the hepatocyte metabolic programme, linking the inflammatory state of the liver to its capacity to adapt to changes in nutrient availability.

The relationship is bidirectional. Hepatic macrophages express succinyl-CoA:3-oxoacid CoA transferase (SCOT) and can oxidize ketone bodies generated by neighbouring hepatocytes, establishing a local hepatocyte–macrophage ketone shuttle [47,74]. Ketone-body utilization may provide macrophages with an alternative oxidative substrate while simultaneously influencing their inflammatory state. Ketogenesis may therefore be viewed not solely as a hepatocyte-autonomous pathway, but as a component of intrahepatic immunometabolic communication. In MASLD, disruption of this macrophage–hepatocyte axis could uncouple lipid availability, ketone production and immune signalling [75], thereby linking impaired ketogenic adaptation to persistent hepatic inflammation and disease progression.

### 5.3. Epigenetic Regulation and Metabolic Reprogramming

One of the most significant conceptual advances in ketone biology has been the discovery that βHB directly regulates chromatin organization and gene expression [76,77,78,79]. βHB links cellular nutrient status to transcriptional adaptation through multiple epigenetic mechanisms (Figure 2).

βHB inhibits class I histone deacetylases (HDACs), thereby increasing histone acetylation and promoting the expression of genes involved in oxidative stress resistance, mitochondrial function and cellular survival [80]. This mechanism provides a direct molecular connection between mitochondrial metabolism and nuclear transcriptional programmes. In parallel, βHB serves as the precursor for lysine β-hydroxybutyrylation, a post-translational histone modification that has emerged as an additional regulator of metabolic gene expression [81]. Although the physiological significance of β-hydroxybutyrylation remains incompletely defined, growing evidence suggests that it coordinates fasting-induced transcriptional responses and cellular adaptation to nutrient stress.

These epigenetic effects are particularly relevant to MASLD, where persistent metabolic overload disrupts transcriptional programmes governing mitochondrial function, lipid metabolism and oxidative stress responses [7]. Reduced βHB production may therefore impair the ability of hepatocytes to mount adaptive gene expression programmes in response to chronic lipid excess. Whether alterations in βHB-dependent chromatin regulation contribute directly to disease progression needs further investigation.

### 5.4. Redox Homeostasis and Mitochondrial Quality Control

Maintenance of mitochondrial integrity is essential for hepatic metabolic homeostasis. Mitochondria not only generate ATP but also coordinate redox balance, ROS signalling, apoptosis, and intermediary metabolism [82]. Progressive mitochondrial dysfunction is a defining feature of MASLD and is closely associated with oxidative stress, impaired oxidative phosphorylation and reduced metabolic flexibility [6].

βHB appears to influence several aspects of mitochondrial biology (Figure 2). Oxidation of ketone bodies generates ATP with favourable energetic efficiency and may reduce oxidative stress under specific metabolic conditions by modifying mitochondrial substrate utilization and redox state [83]. Previous studies further suggest that βHB enhances antioxidant defence systems, promotes mitochondrial biogenesis and improves mitochondrial quality control through regulation of autophagy and mitophagy [25]. These observations raise the possibility that ketogenesis contributes to mitochondrial resilience not only by removing excess acetyl-CoA but also by activating signalling pathways that preserve mitochondrial function during metabolic stress.

Nevertheless, many of these mechanisms remain incompletely understood. It is often difficult to distinguish the direct signalling effects of βHB from secondary metabolic adaptations accompanying increased ketone oxidation or fasting. Furthermore, the concentrations of βHB required to elicit signalling responses in experimental systems frequently exceed those observed in individuals with MASLD. Future studies should use metabolic flux approaches to distinguish endogenous ketogenesis from exogenous ketone supplementation and define how ketone metabolism differs between tissues.

## 6. Therapeutic Implications: Targeting Ketogenic Metabolism to Restore Metabolic Flexibility

### 6.1. Dietary Interventions

Dietary interventions provide the strongest physiological evidence that modulation of ketogenesis influences hepatic metabolism. Caloric restriction, intermittent fasting, time-restricted feeding and carbohydrate restriction all stimulate endogenous ketogenesis while simultaneously improving insulin sensitivity, mitochondrial function and hepatic lipid metabolism [84,85]. Clinical studies consistently demonstrate reductions in liver fat following these interventions [86,87,88], although the relative contribution of ketogenesis remains difficult to distinguish from the effects of weight loss and improved metabolic health. Importantly, ketogenic diets differ from conventional caloric restriction primarily through marked carbohydrate restriction, which lowers insulin signalling, increases adipose lipolysis and hepatic fatty-acid oxidation, and thereby promotes endogenous ketogenesis and βHB production [89]. These effects provide a mechanistic basis by which ketogenic diets could influence hepatic metabolism beyond energy restriction alone. However, whether enhanced ketogenesis independently contributes to reductions in liver fat remains unresolved, as weight loss and improved insulin sensitivity frequently occur concurrently.

Ketogenic diets should not be considered synonymous with therapeutic ketogenesis. Nutritional ketosis represents only one component of the complex metabolic adaptations induced by dietary interventions, which also influence adipose tissue lipolysis, hepatic de novo lipogenesis, gut microbiota composition, bile acid metabolism and endocrine signalling. Therefore, improvements observed with ketogenic diets cannot currently be attributed specifically to increased ketone production. Moreover, prolonged exposure to diets rich in saturated fat may increase hepatic lipid delivery and potentially offset some metabolic benefits. Future studies will need to determine the level and duration of ketogenic activation required to improve hepatic metabolism without causing adverse effects, particularly in advanced MASLD. Isocaloric comparisons of ketogenic and non-ketogenic diets, together with direct measurements of hepatic ketogenic flux, will be important for determining whether ketogenesis itself provides an independent therapeutic benefit.

### 6.2. Pharmacological Modulation of Ketogenic Pathways

Direct pharmacological targeting of ketogenesis remains largely unexplored. Because HMGCS2 represents the rate-limiting enzyme of ketone body synthesis [90], selective enhancement of HMGCS2 expression or activity has attracted considerable interest as a potential therapeutic strategy. However, the complex transcriptional regulation of ketogenesis and its integration with multiple metabolic pathways make isolated manipulation of HMGCS2 unlikely to be sufficient.

Several existing therapies may indirectly restore ketogenic capacity by improving mitochondrial function or reducing metabolic stress. Peroxisome proliferator-activated receptor-α (PPARα) agonists increase fatty acid oxidation and induce expression of ketogenic enzymes [39], whereas fibroblast growth factor 21 (FGF21) analogues enhance mitochondrial metabolism and promote adaptive fasting responses [91]. Similarly, agents that improve insulin sensitivity may alleviate hyperinsulinaemia-mediated suppression of ketogenesis [92]. Whether these therapeutic effects depend, at least in part, on restoration of ketogenic flux remains uncertain and warrants further investigation.

Exogenous ketone supplementation has also emerged as a potential strategy for exploiting the signalling properties of βHB [93]. Ketone esters and ketone salts rapidly increase circulating βHB concentrations without requiring endogenous ketogenesis [94,95]. Although this approach offers an opportunity to dissect the signalling effects of βHB independently of hepatic metabolism, it may not reproduce the broader metabolic adaptations associated with physiological ketogenesis. Consequently, whether exogenous ketones can substitute for endogenous ketogenic function in MASLD remains an open question.

Emerging therapeutic approaches targeting impaired ketogenesis can be broadly classified according to whether they directly enhance endogenous ketone production, indirectly restore ketogenic capacity, or bypass hepatic ketogenesis through exogenous ketone delivery (Table 1). Although several existing metabolic therapies may influence ketogenic pathways, direct pharmacological restoration of hepatic ketogenesis remains largely unexplored.

### 6.3. Integrating Ketogenic Modulation with MASLD Therapies

Several pharmacological therapies are now being developed for MASLD. Glucagon-like peptide-1 (GLP-1) receptor agonists, dual incretin agonists, thyroid hormone receptor-β agonists and agents targeting de novo lipogenesis primarily reduce hepatic lipid burden by improving systemic metabolism or altering substrate availability [96,97,98]. Modulation of ketogenesis is unlikely to replace these approaches but may instead complement them by improving intracellular carbon partitioning and mitochondrial metabolic flexibility. Future therapeutic strategies may therefore require combination approaches that simultaneously reduce lipid influx while enhancing the liver’s capacity to safely process excess fatty acids.

## 7. Knowledge Gaps and Future Directions

Despite increasing recognition of the importance of ketogenesis in hepatic metabolism, several fundamental questions remain unresolved. Addressing these uncertainties will be essential for determining whether ketogenic dysfunction represents a viable therapeutic target in MASLD or simply reflects a secondary consequence of progressive metabolic disease.

Whether impaired ketogenesis initiates metabolic dysfunction or instead develops as a consequence of mitochondrial injury remains unclear. Current evidence supports elements of both models. Suppression of ketogenesis promotes oxidative stress, inflammation and hepatic injury, suggesting a causal contribution [99]. Conversely, progressive mitochondrial dysfunction itself limits ketogenic capacity, indicating that impaired ketogenesis may also arise secondary to established disease [100]. These mechanisms may operate together, with mitochondrial dysfunction and impaired ketogenesis reinforcing each other during disease progression.

Further, the temporal regulation of ketogenesis across the spectrum of MASLD is not fully understood. Most available studies provide cross-sectional measurements of circulating ketone concentrations rather than direct assessments of hepatic ketogenic flux. As ketone body concentrations are determined by both hepatic production and peripheral utilization [11], they provide only an indirect measure of hepatic metabolism. Stable isotope tracer methodologies, metabolomic flux analyses and in vivo imaging approaches will therefore be required to define how ketogenesis changes during different stages of disease progression.

The contribution of different liver cell populations to altered ketogenesis remains poorly understood. Although hepatocytes are responsible for ketone body synthesis, disease progression is determined by complex interactions among hepatocytes, Kupffer cells, infiltrating immune cells, hepatic stellate cells and liver sinusoidal endothelial cells [3]. Whether ketone bodies differentially regulate these cell populations remains largely unknown. Technologies, including single-cell transcriptomics, spatial metabolomics and multiplex imaging, now provide opportunities to investigate how ketogenic signalling shapes the hepatic microenvironment with unprecedented resolution.

The interaction between ketogenesis and other metabolic signalling networks also remains poorly understood. Previous studies suggest extensive crosstalk among ketone metabolism, bile acid signalling, fibroblast growth factor pathways, gut microbiota-derived metabolites and circadian regulation [101,102]. Integrating these pathways into unified models of hepatic metabolic adaptation will likely provide a more comprehensive understanding of MASLD than studying individual pathways in isolation.

Furthermore, lean MASLD, obesity-associated MASLD and diabetes-associated MASLD differ substantially in their metabolic characteristics [103,104,105,106], yet whether ketogenic regulation varies among these phenotypes remains unknown. Similarly, genetic variants that influence hepatic lipid metabolism may modify ketogenic capacity and therapeutic responsiveness [107]. Stratifying patients according to metabolic phenotype instead of histological severity alone may therefore become increasingly important for the development of precision medicine approaches.

## 8. Conclusions

The traditional view of ketogenesis as a fasting-induced pathway for ketone body production no longer fully reflects its biological role. In MASLD, ketogenesis should instead be viewed as a component of hepatic metabolic flexibility that links fatty acid oxidation, mitochondrial carbon disposal and intercellular signalling. Ketogenesis contributes to the regulation of mitochondrial function, lipid metabolism and cellular signalling. Through the production of β-hydroxybutyrate, it also influences inflammatory responses, oxidative stress and gene regulation, placing ketogenesis at the intersection of metabolism and cell signalling.

The evidence reviewed in this study suggests that impaired ketogenic adaptation may contribute to MASLD by uncoupling fatty acid supply from mitochondrial disposal, thereby promoting lipotoxicity, mitochondrial stress, and inflammatory signalling. This metabolic defect is further shaped by insulin resistance, autophagy, lipid-droplet turnover and hepatic immune–metabolic crosstalk, positioning ketogenesis within a broader network that determines how the liver responds to nutrient excess.

The therapeutic implication is not simply to increase ketone-body concentrations, but to determine whether restoring endogenous ketogenic capacity can re-establish hepatic metabolic flexibility and alter disease progression. Defining ketogenic flux in humans and identifying the metabolic contexts in which its restoration is beneficial will be critical for translating this emerging biology into precision therapies for MASLD.

## Figures and Tables

**Table 1:** Emerging therapeutic strategies targeting or bypassing impaired hepatic ketogenesis in MASLD.

Therapeutic Approach	Main Mechanism	Potential Role in Impaired Ketogenesis	Refs.
Dietary interventions	Caloric restriction, intermittent fasting, time-restricted feeding and carbohydrate restriction stimulate fatty-acid oxidation and endogenous ketogenesis	Enhance physiological ketogenesis while improving insulin sensitivity, mitochondrial function and hepatic lipid metabolism	[84,85,86,87,88,89]
HMGCS2 modulation	Targeting HMGCS2, the rate-limiting enzyme of ketone-body synthesis	Directly enhance hepatic ketogenesis and potentially restore ketogenic capacity	[90]
PPARα agonists	Increase fatty-acid oxidation and expression of ketogenic enzymes	Indirectly enhance hepatic ketogenic capacity	[39]
FGF21 analogues	Promote mitochondrial metabolism and adaptive fasting responses	Potentially restore ketogenic capacity and metabolic flexibility	[91]
Insulin-sensitizing therapies	Reduce hyperinsulinaemia-mediated suppression of ketogenesis	Indirectly restore endogenous ketone production	[92]
Exogenous ketones (ketone esters/salts)	Increase circulating β-hydroxybutyrate independently of hepatic ketogenesis	Bypass impaired endogenous ketogenesis and exploit βHB signalling	[94,95]
GLP-1 receptor agonists and dual incretin agonists	Improve systemic metabolism and reduce hepatic lipid burden	May complement ketogenic modulation by improving substrate availability and metabolic flexibility	[96,97,98]
Thyroid hormone receptor-β agonists/DNL-targeting agents	Reduce hepatic lipid accumulation through altered hepatic lipid metabolism	Potential complementary strategies to enhance the metabolic benefits of restored ketogenic capacity	[96,97,98]

## Data Availability

No new data were created or analyzed in this study. Data sharing is not applicable to this article.

## Abbreviations

MASLD	Metabolic dysfunction-associated steatotic liver disease
NAFLD	Non-alcoholic fatty liver disease
MASH	Metabolic dysfunction-associated steatohepatitis
VLDL	Very low-density lipoprotein
βHB	β-hydroxybutyrate
NASH	Non-alcoholic steatohepatitis
TCA	Tricarboxylic acid
ATP	Adenosine triphosphate
PPARα	Proliferator-activated receptor-α
HMGCS2	3-hydroxy-3-methylglutaryl-CoA synthase 2
FOXA2	forkhead box protein A2
HNF4α	Hepatocyte nuclear factor 4 alpha
PGC-1α	Peroxisome proliferator-activated receptor gamma coactivator 1-alpha
ROS	Reactive oxygen species
DNA	Deoxyribonucleic acid
TGF-β	transforming growth factor-β
NLRP3	NLR family pyrin domain containing 3
IL-1β	Interleukin-1β
IL-18	Interleukin 18
TNF-α	Tumour necrosis factor-alpha
SCOT	Succinyl-CoA:3-oxoacid CoA transferase
HDAC	Histone deacetylase
FGF21	Fibroblast growth factor 21
GLP1	Glucagon-like peptide-1
